# Inflammatory Determinants and Associated Morbidity in Hemodialysis Patients

**DOI:** 10.3390/jpm13091311

**Published:** 2023-08-27

**Authors:** Claudia Jackelin De la Cruz-Ahumada, Jorge Fernando Topete-Reyes, Juan Pablo Mena-Ramírez, Juan Manuel Guzmán-Flores, Jesúa Ivan Guzmán-González, Saúl Ramírez-De los Santos

**Affiliations:** 1 Laboratorio de Investigación en Biociencias, Centro Universitario de los Altos, Universidad de Guadalajara, Tepatitlán de Morelos 47620, Jalisco, Mexico; claudia.delacruz@academicos.udg.mx (C.J.D.l.C.-A.);; 2 Instituto Mexicano del Seguro Social, HGR 46, Guadalajara 44910, Jalisco, Mexico; 3 Instituto Mexicano del Seguro Social, HGZ 21, Tepatitlán de Morelos 47639, Jalisco, Mexico; 4 Departamento de Psicología Básica, Centro Universitario de Ciencias de la Salud, Universidad de Guadalajara, Guadalajara 44340, Jalisco, Mexico

**Keywords:** associated morbidity, cytokines, hemodialysis, inflammatory determinants

## Abstract

Hemodialysis deteriorates patients’ physical, metabolic, and mental status. Clinical outcomes derived from inflammation determine a worse status but are less frequently identified. The objective of the study was to identify inflammatory determinants and the effect of SNP-related serum IL-6 and IL-10 levels on associated morbidity in hemodialysis. A sample of hemodialysis patients at IMSS Regional Hospital No.46 in Guadalajara (*n* = 85) were tested using the Malnutrition Inflammation Score (MIS) and Patient Health Questionnaire-9 (PHQ-9) to assess the associated morbidity. Serum cytokine levels were quantified by enzyme-linked immunosorbent assay (ELISA). The restriction fragment length polymorphism (RFLP) technique was used for analysis of *IL-6*-572C/G and *IL-10*-1082A/G. Using data visualization methods, we identified relevant determinants of inflammation. A simple regression model was constructed between predictors and targets with genotypes as covariates. Results showed malnutrition in 85.9% of patients and depressive symptoms in 50.6%. IL-10 was the most relevant inflammatory determinant, with regression coefficients (R^2^) between 0.05 and 0.11. The GG genotype of *IL-10*-1082 A/G evinced small effect on both clinical outcomes (δ of 0.35 and 0.37, respectively). Hemodialysis increases the associated morbidity, cytokines act as inflammatory determinants, and genetic variability contributes to the severity of clinical outcomes. Further studies need to refine the causal relationship between inflammation and CKD.

## 1. Introduction

Worldwide, chronic kidney disease (CKD) affects eight to sixteen percent of the population. It is a leading cause of morbidity and mortality, and this implies a high social burden [1]. CKD greatly impairs individuals’ quality life, especially in those with the most advanced stages or who are on replacement therapy [2]. Hemodialysis continues to be the most common form of replacement therapy [3]; despite its advantages, hemodialysis entails a reduced adherence to treatment, poor eating habits, and low physical activity [4], which in turn further impair the patient’s physical, functional, metabolic, social, and mental status [4,5]. In addition, adverse therapeutic effects may complicate the clinical picture.

Patients experience negative affective moods, impotence in coping with the disease, and poor adjustment to replacement therapy, which alters physiological and behavioral patterns [6,7]. Apart from its negative impact on the individual’s well-being, hemodialysis activates the immune response through dendritic cells and the consequent release of inflammatory cytokines [8] that ultimately account for various clinical signs such as depression and protein-energy wasting [9,10]. Although the clinical outcomes derived from inflammation and the treatment’s adverse effects determine a worse prognosis, they are identified and treated less frequently than other disease factors [11].

Inflammation and cardiovascular disease might influence the development of depression [12], the mental disorder most prevalent in CKD [12,13], which is exacerbated in patients undergoing hemodialysis when coexisting with conditions such as malnutrition and sarcopenia [14]. Hemodialysis is today’s treatment option for end-stage kidney disease, and although it has continued to progress, the patients face a high risk of malnutrition, which is one of the strongest predictors of morbidity and mortality for them [15,16]. For healthcare professionals, early diagnosis and intervention strategies for these clinical outcomes must be a priority to improve the physical and mental well-being of this vulnerable population.

CKD patients have increased pro-inflammatory cytokine production and increased oxidative stress, leading to an inflammatory microenvironment contributing to increased disease morbidity and mortality [16]. Inflammatory cytokine levels vary among hemodialysis patients according to disease stage, as their predictor value is different depending on the disease severity [17]. It is currently unclear which biomarkers offer predictably superior performance for assessing clinical conditions associated with inflammation [16], such as Brys et al. who reported that IL-6 has a stronger predictive value than the C-reactive protein (CRP) in advanced stages [18]. Other studies suggest that inflammatory cytokines contribute to a worse clinical outcome in the advanced stages of CKD [19]. In patients receiving hemodialysis, IL-6 is positively correlated with depression scores and malnutrition but negatively correlated with serum albumin concentrations [20,21,22]. 

In contrast, research involving patients on substitution therapy reported that lower levels of IL-10 were associated with depressive symptoms [23]. A recent meta-analysis of end-stage CKD [24] found that subjects with depressive symptoms had a pattern of elevated proinflammatory IL-6 and low concentrations of anti-inflammatory IL-10. These authors ascribed the discrepant results between studies to heterogeneity in the analyses and to some individuals’ previously observed genetic proneness to an increased inflammatory response [23]. Hence, it is necessary to carry out research that focuses on these variables.

CKD progresses in different ways among individuals, and these differences might not be explained by clinical conditions in patients, suggesting that the genetic component is important [25]. Moreover, there is evidence that genetic variability mediates the inflammatory response [26] and that specific gene variations directly or indirectly affect renal function [27]. Numerous genes have been recognized that when combined with environmental factors, impact CKD [28]. It has been reported that single nucleotide polymorphisms (SNPs) on genes coding for pro- and anti-inflammatory cytokines appear to be associated with susceptibility to CKD [29]; patients may also present a genotypically determined tendency to higher or lower cytokine production, and this thus alters their clinical phenotype [25]. For these reasons, we assume that SNPs in genes involved in the inflammatory response influence CKD progression to some degree.

This research aimed to identify both the inflammatory determinants based on the estimation of relevance and the effect of serum concentrations of cytokines IL-6 and IL-10 related to the SNPs *IL-6 -*572 G/C and *IL-10* -1082 A/G on the associated morbidity in patients receiving hemodialysis. 

## 2. Materials and Methods

### 2.1. Participants and Procedure

The cross-sectional study was performed at the Hospital Regional No. 46 of the IMSS in Guadalajara, Jalisco. It enrolled subjects from Western Mexico diagnosed with stage 5 CKD according to the KDIGO guidelines and who attended their morning or evening hemodialysis sessions during October 2022. Patients with active infectious diseases, immunodeficiencies, cancer, morbid obesity, or any neuropsychiatric disease were omitted. Those who did not complete the questionnaires or whose sample was lost were excluded. Potential participants received an information form describing the aim of the research. Then, those who agreed to participate provided their written informed consent before the application of the questionnaires and the blood collection. The Bioethics Committee (ACTA: CEI-01-2022-001) and the Biosafety Committee (ACTA: CBIO-01-2022-01) of the Centro Universitario de los Altos of the Universidad de Guadalajara approved the project.

### 2.2. Measures

#### 2.2.1. Cytokine Serum Levels

A 4-mL peripheral blood sample was collected in EDTA vials through the hemodialysis vascular line at the beginning of the treatment session and was subsequently used to determine IL-10 and IL-6 levels. Serum concentrations of the cytokines were quantified by enzyme-linked immunosorbent assay (ELISA) kits (Human IL-10 Duo Set^®^ ELISA DEVELOPMENT SYSTEM Cat. Number DY217B-05 and Human IL-6 ELISA MAXTM Deluxe Set Cat. Number 430504). The assay procedure was performed as instructed by the manufacturer. The result of cytokine levels was expressed in picograms per milliliters (pg/mL).

#### 2.2.2. Malnutrition Inflammation Score (MIS)

The Malnutrition Inflammation Score (MIS), which consists of anthropometric measurements, biochemical data, and subjective global assessment (SGA) components, was used to assess nutritional status. The questionnaire consists of five sections: medical history, dietary intake, physical examination, body mass index (BMI), biochemical parameters, and total iron binding capacity (TIBC) or transferrin; each MIS component is assigned to one of four severity levels ranging from 0 (normal) to 3 (very severe). The total score is the sum of the 10 components assessed and ranges from 0 to 30. It was categorized as follows: normal nutritional status: 0, 1, and 2 points; mild malnutrition: 3–5; moderate malnutrition: 6–8; severe malnutrition: from 9 points.

#### 2.2.3. Patient Health Questionnaire-9 (PHQ-9)

The Patient Health Questionnaire-9 (PHQ-9) was used for screening depressive symptoms and their severity. The PHQ-9 consists of 9 questions that are based on the 9 DSM-IV criteria for major depressive disorder. The questionnaire explores the symptoms experienced by patients during the immediately preceding 2 weeks. Scores for each item on the PHQ-9 range between 0 (not at all), 1 (several days), 2 (more than half of the days), and 3 (almost every day). The summed scores range from 0 to 27. Each score was classified as follows: 0 to 4 points, absence of depressive symptoms; 5 to 9 points, minimal symptoms; 10 to 14 points, minor depressive symptoms; 15 to 19 points, moderate major depressive state; and >20 points, severe major depression according to the manual of the questionnaire.

#### 2.2.4. Genotypes

We extracted blood genomic DNA with the PureLink^®^ Genomic DNA Mini Kit Cat. Number K1820-01 and NucleoSpin^®^Blood Mini Kit. DNA samples were stored at −80 °C until used to analyze the single nucleotide polymorphism (SNP) *IL-6*-572C/G (db SNP ID rs1800796) and *IL-10*-1082A/G (db SNP ID rs1800896). We searched these SNPs by coupling the polymerase chain reaction (PCR) with the restriction fragment length polymorphism (RFLP) technique.

For the *IL-6*-572G/C variant, we amplified a 163-bp fragment using two primers: direct 5′-GGA GAC GCC TTG AAG TAA CTG C-3′ and inverse 5′-GAG TTT CCT CTG ACT CCATCG CAG-3. For the *IL-10*-1082A/G variant, the amplified fragment was 102 bp long while the primers were 5′AACACTACTAAGGCTCCTTTGGGA-3′ (direct) and 5′-CAAGGAAAAGAAGTCAGGATTCCATGGA-3 (inverse). The 20 µL reaction mixture contained 10 µL of BioMixTM Red plus (dNTPs, reaction buffer and Taq DNA), 0.5 µM of each primer and 3 µL of DNA for *IL-6*-572C/G or 0.2 µM of each primer and 6 µL of DNA for *IL-10*-1082A/G; nuclease-free water was added to complete 20 µL. After initial denaturation at 95 °C for 5 min, 37 PCR cycles were performed: 30 s at 95 °C, 45 s at 55.2 °C, and 1 min at 72 °C, with a final extension at 72 °C for 7 min.

Then, 10 µL of the PCR products were digested overnight at 37 °C in a dry bath with 1 U of BsrBI restriction enzyme for *IL-6*-572G/C or 0.5 U of EcoNI restriction enzyme for *IL-10*-1082A/G. Digested PCR products were electrophoresed on 2% agarose gel and viewed by ultraviolet irradiation after SYBR*Safe staining, applying positive controls. The electrophoretic pattern for *IL-6*-572G/C was GG, 102 and 61 bp; GC, 163, 102, and 61 bp; and CC, 163 bp fragment. For *IL-10*-1082A/G, it was AA, 102 bp; AG, 102, 82, and 20 bp; and GG, 82 and 20 bp.

### 2.3. Data Analysis

Statistical analysis was performed using Jamovi 2.3.21 version. For the descriptive analysis, we evaluated the variables using frequency and percentage distribution tables while for cytokine levels we used medians (interquartile range) given the nonparametric distribution. Using a model based on the CIBER (Confidence Interval Based Relevance Estimation) method [30], we identified the determinants of inflammation in malnutrition–inflammation and depressive symptomatology. We determined the association of genotypes with cytokine levels on the associated morbidity using a simple regression model between predictors and targets, visualized with a flex plot analysis [31].

## 3. Results

### 3.1. Demographic and General Characteristics

Complete data were obtained from 85 individuals, including 47 (54%) men, with a mean age of 41 ± 15 years. About half of them (49.4%) perceived their health status as regular; renal hypoplasia was the leading cause of CKD (41.2%). The mean subject’s weight was 63.8 ± 17.3 kg and 48.2% of participants had normal weight by body mass index (BMI). Although the mean systolic and diastolic blood pressure (BP) was 137.9 mmHg and 78.6 mmHg, over half of the individuals (65.9%) were classified in some category of elevated BP. Table 1 presents the demographic and general characteristics of the participants.

### 3.2. Serum Levels of IL-6 and IL-10

Serum levels of both cytokines were expressed as medians (interquartile range) and were classified into two groups (Table 2: out of range (quartiles 1 and 4) and in range (quartiles 2 and 3). IL-6 had a median of 37.33 (0.00–258.17) pg/mL with out-of-range values (44.0–269.8) in 75% of patients, while IL-10 had a median of 22.33 (0.00–141.50) pg/mL with out-of-range concentrations (22.3–141.5) in 71.8% of the patients.

### 3.3. Associated Morbidity

Based on the individuals’ responses to the MIS and PHQ-9 questionnaires, we categorized the variables malnutrition–inflammation and depressive symptomatology by the sum of the items. For the latter, the mean score was 6.7 ± 7.1. Table 3 shows the frequency of associated morbidities. According to MIS and PHQ-9 scores, 85.9% (73) of the subjects had some degree of malnutrition and 50.6% (43) reported minimal to severe depression. The frequency of the out-of-range levels for both cytokines and the most prevalent genotype for either SNP in each category of clinical outcome are also described.

### 3.4. Inflammatory Determinants

**Figure 1:**
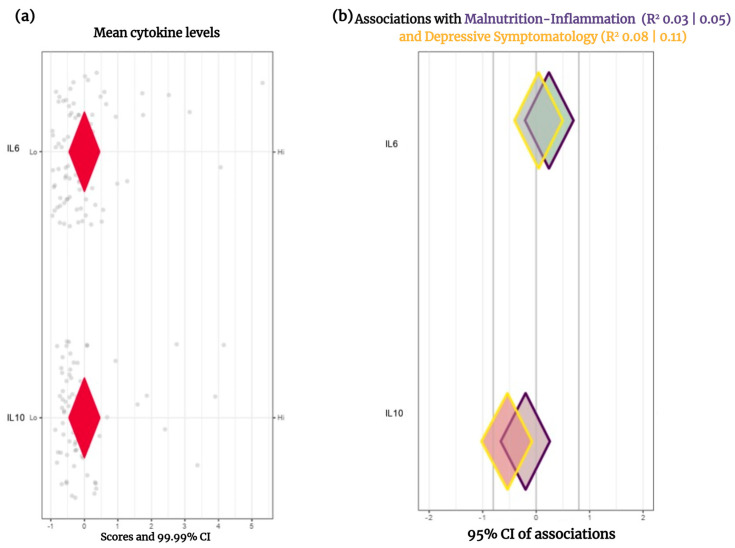
Inflammatory determinants on Malnutrition–Inflammation and Depressive Symptomatology. (**a**) Means are presented as Z-scores (the redder the diamond is, the lower the mean concentration is). (**b**) The color of the diamond’s line differs between clinical outcomes. The diamond’s fill color indicates the strength of the association and its direction (the redder the diamonds are, the stronger the negative associations are. The greener the diamonds are, the stronger the positive associations are).

Using the CIBER method, we identified that the most relevant determinant of malnutrition–inflammation and depressive symptomatology was IL-10 with coefficients (R^2^) of 0.05 and 0.11, respectively (Figure 1). 

### 3.5. Association of Genotypes and Cytokine Levels at Clinical Outcome

To identify the association of genotypes with cytokine levels on malnutrition–inflammation severity and depressive symptomatology, we built a simple regression model between predictors (cytokines) and targets (MIS and PHQ-9 scores) where such genotypes were covariates. 

For depression symptom severity, the C allele of *IL-6*-572 G/C showed no effect on the PHQ-9 score (GC–GG, δ = 0.1), meanwhile the GG genotype of *IL-10*-1082 A/G exhibited a small effect on symptom severity (GG–AA, δ = −0.35). The effect size by genotype for the PHQ-9 score is detailed in Table 4.

The C allele of *IL-6*-572 G/C presented a small effect (GC–GG, δ = 0.27) on the MIS score; in contrast, the GG genotype of *IL-10*-1082 A/G showed a small effect on malnutrition–inflammation severity (GG–AA, δ =0.37). The same was true of the G allele (AG–AA, δ = 0.33). The effect size by genotype for the MIS score is described in Table 5.

**Figure 2:**
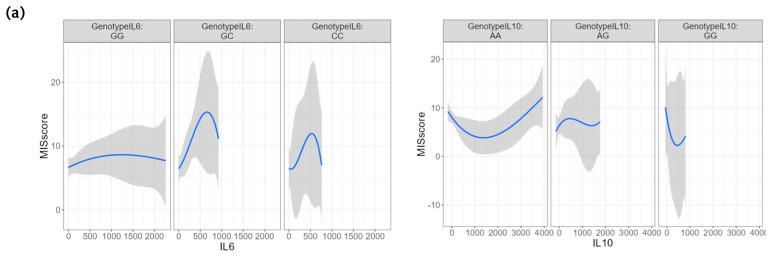

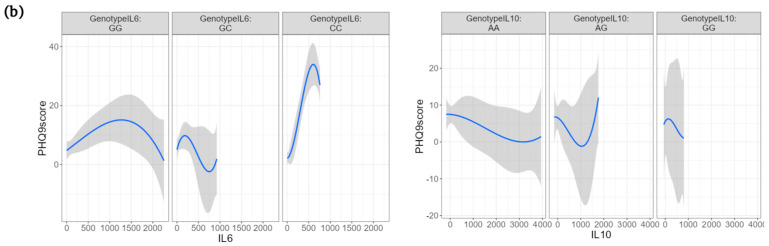
**Cytokine serum concentration and MIS and PHQ-9 scores, by genotype.** (**a**) Added variables plot for the bivariate relationship of IL-6 and IL-10 with MIS score for every *IL-6*-572 G/C and *IL-10*-1082 A/G genotype. (**b**) Added variables plot for the bivariate relationship of IL-6 and IL-10 with PHQ-9 score for every *IL-6*-572 G/C and *IL-10*-1082 A/G genotype.

The added variables plot (AVP) visualizes the G allele of *IL-6*-572 G/C presenting the greatest upward effect on both scores, while the GG genotype (homozygous with the variant) of *IL-10*-1082 A/G had the greatest downward effect on both scores (Figure 2). 

## 4. Discussion

Since replacement therapy has increased the survival rate of individuals in a terminal stage of CKD, further research should evaluate their quality of life and factors that increase morbidity and mortality [5]. This study identified hemodialysis as exerting a certain impact on the nutritional and emotional status of individuals and triggering the inflammatory cytokine release that determines the severity of clinical outcomes.

Low-grade inflammation in CKD is a reliable predictor of the associated morbidity and mortality [32]. Hemodialysis prompts a blood–dialyzer interaction that activates the immune response by releasing cytokines, including IL-6 and IL-10 [33,34]. Therefore, it is necessary to assess the levels of these inflammatory biomarkers for adequate management of the disease. 

Using the CIBER model that advantageously combines several metrics (correlation coefficients, means, and confidence intervals), we identify IL-10 as the major inflammatory determinant of associated morbidity. In our work, the severity of depressive symptoms was explained by the 11% maximum from the IL-10 low serum levels. According to reports, the relationship between inflammation and depression runs in both directions: some research has described depression as leading to an inflammatory state, while other studies conclude inflammation as a factor for depressive symptoms, and a study with a follow-up of two years established a bidirectional relationship [35]. These findings highlight a need for further research to elucidate the causal relationship between inflammation and depression, as mechanisms implicating IL-10 are unclear. 

Results concerning the IL-10 association with depression in CKD are inconsistent. Some studies have reported IL-10 levels to be similar in subjects with or without depression [36]; in contrast, a negative correlation between depression score and IL-10 serum concentration was identified through univariate analysis [37]. Meanwhile, Taraz et al. reported a positive association between the IL-6 to IL-10 ratio and the risk of depression [38]. This is consistent with the findings in our analysis of inflammatory determinants.

The association of high IL-6 levels with the severity of depressive symptomatology indicates that both an impaired immune response and inflammation have a role in the onset and development of depressive symptoms in CKD [2,39]. A meta-analysis of inflammatory biomarkers, including IL-6, in CKD subjects supported the association of such markers with the presence and severity of depressive symptoms [40].

In this study, we used the analysis based on visualization data, one of several new methods developed for exploring genotype effects in linear models [41], to gauge the effectiveness of certain gene variants and cytokine serum levels on associated morbidity in patients receiving hemodialysis. Our findings agree with those of Holtzman et al. who reported that patients receiving hemodialysis carrying the G allele of the *IL-10*-1082 A/G variant showed less severe depressive symptoms [23]. Likewise, the G allele was associated with low levels of inflammatory biomarkers in patients receiving hemodialysis [42]. 

Given the IL-10 immunoregulatory function, its expression maintains the balance with proinflammatory cytokines. Yet, deteriorated kidney function increases the IL-10 blood concentration [17], though it is unclear whether a higher concentration is associated with a better or worse prognosis [27]. It is known that IL-10 reduces the adverse effects of proinflammatory cytokines on neuroplasticity by blocking the detrimental effects of lipopolysaccharides (LPS) or IL-1β and IL-6. Furthermore, this cytokine might have a direct effect on microglia activation, decreasing neuroinflammation [43]. This is in agreement with the findings about a genetic predisposition to a lower inflammatory response that increases the risk for symptoms of depression [23]. 

Regarding the role of the *IL-6*-572 G/C variant as a susceptibility marker in inflammatory diseases [44,45,46], we identified that the CC genotype was related to a higher depressive symptomatology score and the heterozygous genotype (CG) was related to higher scores in the assessment of malnutrition–inflammation. In a Mexican population, Martínez-Ramírez et al. reported a risk association of diabetes mellitus 2 (DM2) and uncontrolled biochemical parameters with the CC and GC genotypes [46]. In contrast, an increased inflammatory susceptibility in individuals with DM2 carrying the GG genotype supports the contention that gene–cytokine interactions are implicated in chronic inflammation and increased morbidity [47].

The association observed here between IL-6 levels and the severity of depressive symptomatology confirms that alterations in immune response and inflammation contribute to the development of depressive symptoms in CKD [2,39]. In addition, IL-6 deteriorates the nutritional status [8], which adversely affects individuals’ mental health. The mechanisms underlying malnutrition due to inflammation in CKD are multifactorial. Proinflammatory cytokines, such as IL-6, induce anorexia and increase the incidence of chronic fatigue and muscle protein degradation. In addition, depression induced by IL-6 can result in a reduction in food intake, an increase in resting energy expenditure, and a suppression of anabolic hormones such as growth hormone and insulin-like growth factor (IGF)-1 [16].

Kidney structural damage produces inflammatory cytokines that reach the vagus nerve’s afferent pathway, which transmits inflammatory signals to the medulla oblongata [48]. Furthermore, peripheral stimuli such as psychological stress and infections trigger microglia activation and cytokine release, which subsequently induce macrophage and cytokine entry into the brain [39,49]. Neurological effects due to the sustained activity of inflammatory cytokines include signaling, synthesis, reuptake, and the release of neurotransmitters [22,50]. Therefore, the underlying biological mechanism likely includes alterations in neurotransmitter function and cortisol elevation linked to stimulation of the hypothalamic–pituitary–adrenal (HPA) axis [8].

The main weaknesses of our research are its cross-sectional design and the lack of follow-up with the patients to assess how their symptoms and nutritional status evolve. Additionally, the inflammatory biomarkers we evaluated might not be enough to determine the interrelationships’ complexity. 

Despite these limitations, the present research showed that the inflammatory response of patients receiving hemodialysis and inter-individual differences secondary to gene variants influence the severity of clinical outcomes. Our work also illustrates the importance of screening nutritional, psychological, and immunological factors of CKD to improve therapeutic management.

## 5. Conclusions

The increased prevalence of malnutrition–inflammation and depressive symptomatology here observed agrees with the highly associated morbidity documented in individuals with CKD receiving hemodialysis. Moreover, we identified IL-10 as an inflammatory determinant for associated morbidity and that *IL-6*-572 G/C and *IL-10*-1082 A/G SNPs contribute to the severity of malnutrition–inflammation and depressive symptoms.

Our findings support the claim that the interaction between traditional and non-traditional factors increases long-term morbidity and mortality in patients undergoing hemodialysis. Accordingly, we suggest a more extensive evaluation of nutritional, psychological, and neuroimmune parameters as well as the multidisciplinary management of CKD integrating behavioral interventions, targeted nutritional therapies, and an assessment of biomarkers of inflammation to prevent or delay the disease’s progression. Lastly, prospective studies that include more clinical, psychological, endocrine, and immunological parameters of CKD at various stages must refine the causal relationship between inflammation and associated morbidity in CKD.

## Figures and Tables

**Table 1:** Sociodemographic and general characteristics of the participants.

Variable	*n*	%	Mean	DE
Gender				
Male	46	54		
Female	39	46		
Age (years)			41	15
Health Perception				
Poor	6	7.1		
Regular	42	49.4		
Good	33	38.8		
Very good	4	4.7		
CKD Causes				
Diabetes Mellitus	10	11.8		
Hypertension	1	1.2		
Polycystic disease	3	3.5		
Hypoplasia renal	35	41.2		
Other	6	7.1		
Not identified	30	35.3		
Weight (kg)			63.8	17.3
BMI (kg/m^2^)			24.1	5.7
Underweight	9	10.6		
Normal weight	41	48.2		
Overweight	22	25.9		
Obese	12	14.1		
BP (mmHg)				
Systolic			137.9	26.8
Diastolic			78.6	14.8
Normal	27	31.8		
Normal High	22	25.9		
Hypertension Grade 1	17	20		
Hypertension Grade 2	17	20		

BMI: Body mass index. BP: Blood pressure.

**Table 2:** Cytokines’ serum levels.

	IL-6 (pg/mL)	IL-10 (pg/mL)
	37.33 (0.00–258.17)	22.33 (0.00–141.5)
Out of range	60 (75)	56 (71.8)
In range	20 (25)	22 (28.2)

Out of range: Q1 and Q4. In range: Q2 and Q3. Note: data are presented as medians (interquartile range) for non-normally distributed data. The numbers in the table are frequencies and the numbers in brackets are percentages.

**Table 3:** Frequency of associated morbidity, out-of-range cytokine levels, and most prevalent genotypes.

Clinical Outcomes		Cytokines Out of Range		Most Prevalent Genotype	
		IL-6	IL-10	*IL-6*	*IL-10*
Malnutrition–Inflammation					
Normal	12 (14.1)	4 (66.7)	5 (83.3)	GG (50.0)	AA (66.7)
Mild malnutrition	12 (14.1)	15 (75)	15 (75)	GG (55-0)	AA (50.0)
Moderate malnutrition	31 (36.5)	21 (80.8)	19 (73.1)	GG (48.1)	AA (70.4)
Severe malnutrition	30 (35.3)	20 (71.4)	17 (65.4)	GG (51.6)	AA (67.7)
Depressive symptomatology					
No depressive symptoms	42 (49.4)	33 (80.5)	33 (80.5)	GG (51.2)	AA (68.3)
Minimal symptoms	21 (24.7)	13 (68.4)	12 (66.7)	GG (52.4)	AA (57.1)
Minor depressive symptoms	9 (10.6)	4 (50.0)	4 (50.0)	GG (33.3)	AA (44.4)/AG (44.4)
Moderate major depressive state	8 (9.4)	6 (85.7)	4 (57.1)	GG (62.5)	AA (62.5)
Severe major depression	5 (5.9)	4 (80.0)	3 (75.0)	GG (60.0)	AA (100.0)

Note: the numbers in the table are frequencies and the numbers in brackets are percentages.

**Table 4:** Estimates and effect sizes for categorical predictors for depressive symptomatology.

Variable	Genotype	Estimate	95% Confidence Interval	
			Lower	Upper
Factors (estimates reported are means)				
GENOTYPE *IL-6*	GG	6.71	5.41	8.02
	GC	7.72	6.32	9.12
	CC	6.79	4.62	8.95
Numeric variables (estimates reported are slopes/intercepts)				
Intercept		6.986	5.79	8.18
Slope: IL-6		0.695	−0.15	1.54
Factors (estimates reported are means)				
GENOTYPE *IL-10*	AA	7.67	6.56	8.78
	AG	6.41	4.69	8.13
	GG	6.27	3.44	9.11
Numeric variables (estimates reported are slopes/intercepts)				
Intercept		7.6878	6.66	8.71
Slope: IL-10		0.0492	−0.8	0.9

**Table 5:** Estimates and effect sizes for categorical predictors for malnutrition–inflammation.

Variable	Genotype	Estimate	95% Confidence Interval	
			Lower	Upper
Factors (estimates reported are means)				
GENOTYPE *IL-6*	GG	5.63	3.16	8.1
	CG	6.35	3.69	9.01
	CC	6.28	2.17	10.39
Numeric variables (estimates reported are slopes/intercepts)				
Intercept		6.28	4.02	8.54
Slope: IL-6		1.65	0.04	3.25
Factors (estimates reported are means)				
GENOTYPE *IL-10*	AA	7.38	5.35	9.41
	AG	6.55	3.4	9.71
	GG	4.95	−0.25	10.15
Numeric variables (estimates reported are slopes/intercepts)				
Intercept		6.82	4.94	8.7
Slope: IL-10		−1.58	−3.13	−0.02

## Data Availability

The data that support the findings of this study are available from the corresponding author upon reasonable request.
